# Rehabilitation models that support transitions from hospital to home for people with acquired brain injury (ABI): a scoping review

**DOI:** 10.1186/s12913-023-09793-x

**Published:** 2023-07-31

**Authors:** Marianne Eliassen, Cathrine Arntzen, Morten Nikolaisen, Astrid Gramstad

**Affiliations:** 1 Department of Health and Care Sciences, University of Tromsø, The Artic University of Norway, Tromsø, 9037 Norway; 2 Center for Care Sciences, North, University of Tromsø, The Artic University of Norway, Tromsø, 9037 Norway

**Keywords:** Rehabilitation, Acquired brain injury, Health care services, Early supported discharge, Care trajectory, Transitional care

## Abstract

**Background:**

Research shows a lack of continuity in service provision during the transition from hospital to home for people with acquired brain injuries (ABI). There is a need to gather and synthesize knowledge about services that can support strategies for more standardized referral and services supporting this critical transition phase for patients with ABI. We aimed to identify how rehabilitation models that support the transition phase from hospital to home for these patients are described in the research literature and to discuss the content of these models.

**Methods:**

We based our review on the “Arksey and O`Malley framework” for scoping reviews. The review considered all study designs, including qualitative and quantitative methodologies. We extracted data of service model descriptions and presented the results in a narrative summary.

**Results:**

A total of 3975 studies were reviewed, and 73 were included. Five categories were identified: (1) multidisciplinary home-based teams, (2) key coordinators, (3) trained family caregivers or lay health workers, (4) predischarge planning, and (5) self-management programs. In general, the studies lack in-depth professional and contextual descriptions.

**Conclusions:**

There is a wide variety of rehabilitation models that support the transition phase from hospital to home for people with ABI. The variety may indicate a lack of consensus of best practices. However, it may also reflect contextual adaptations. This study indicates that health care service research lacks robust and thorough descriptions of contextual features, which may limit the feasibility and transferability to diverse contexts.

**Supplementary Information:**

The online version contains supplementary material available at 10.1186/s12913-023-09793-x.

## Background

Acquired brain injury (ABI) is defined as an acute neurological insult that involves a number of conditions and etiologies, such as traumatic brain injury, cardiovascular accident, and hypoxia, among others [1, 2]. The consequences are complex and often multifaceted and can involve motor, cognitive, behavioral, communicative, and emotional challenges [3–5], in addition to changes in personality [6], which may reduce everyday life functioning and quality of life and limit social and societal participation [3–5].

The diversity of etiology and extent of injury in ABI result in outcomes ranging from mild and moderate to severe. Studies have shown that a great proportion of people suffering from either stroke [7] or ABI in general [8] are being discharged directly to their home. For example, approximately four in 10 stroke patients are discharged home without service offerings at all [9, 10]. However, these patients may suffer from undiscovered functional changes that may later have a substantial impact on their everyday lives and well-being [11]. Hence, this scoping review focuses on services for people with ABI classified as mild to moderate who are discharged home.

People with ABI often depend on adequate rehabilitation services due to different and changing needs during the rehabilitation trajectory [1, 12, 13]. Recent changes in the organization of health care services worldwide have led to earlier hospital discharge, and there has been a gap in service delivery between in-hospital rehabilitation and home-based rehabilitation in the community [10, 13]. The transition across different health care levels requires a coordinated trajectories from hospital care to home-based rehabilitation services in the community. However, research that have explored services for stroke patients, who constitute a large portion of persons with ABI, has criticized services for not achieving seamless trajectories that equip patients and carers to cope with the long-term effects of brain injuries [11, 12, 14–16]. Research shows that the transition from hospital to home can be challenging, both for patients [8, 16–19] and for their family caregivers [18, 20].

To close the gap between specialized rehabilitation in hospitals and home-based rehabilitation in primary care, strategies that support early discharge have been suggested [5, 8, 21, 22]. However, descriptions of the content, contexts and organization of such models are diverse, and there are no common guidelines for the discharge phase for patients with ABI [9]. Research also shows that health care providers appear to consider various factors when making referral decisions and admission to follow-up. Variables such as age, mental capacity, and type of injury influence clinicians’ decisions of admission to rehabilitation facilities [23]. Therefore, there is a need to gather and synthesize knowledge about services that can support strategies for more standardized referral for patients with ABI.

Access to rehabilitation varies globally. There are differences in available resources, and there is great variation in the content and organization of service delivery [24]. Many patients and their carers need follow-up after discharge. However, how this can best be delivered remains unknown. Services that support children with ABI may vary to a great extent from services for adult patients and will require separate procedures for assessing the research. In this article, we focus on rehabilitation services for adult patients with ABI (> 18 years). There is a tendency in the research literature to focus more on the outcome of interventions than explaining the content of services. Hence, in this paper, we apply a scoping review methodology to investigate a broader body of the literature that may include more robust descriptions of the content and contextual features of rehabilitation models, such as organization forms, the agents involved, the settings they are applicable in, and any accompanying theoretical rationale.

‘Rehabilitation model’ is a quite ambiguous term, often used without any clear definition or explanation. In this scoping review, we aimed to include a broad view of the research field and have considered a ‘model’ in line with the definition of Bukve [25] as a ‘simplified and systematic representation of certain aspects of the world’ [authors translation]. To grasp a wide aspect of relevant studies, we have used a search strategy that included relevant terms such as ‘framework’, ‘program’, and ‘services.’

The critical transition phase from hospital to home is in interest, as patients and their family members tend to report on challenges concerning this particular phase of rehabilitation [16–18, 20]. Therefore, this scoping review only includes studies that report on rehabilitation starting less than 6 months after the injury.

The purpose of this study was to identify how rehabilitation models that support the transition phase from hospital to home for people discharged with ABI are described in the research literature and to discuss the content of the models.

## Methods

A scoping review is recommended when the purpose is to identify knowledge gaps, scope a body of literature, clarify concepts or investigate research conduct. Levac et al. [26] recommends utilizing scoping studies in fields where the evidence is emerging, such as in rehabilitation. Scoping reviews are ideal, as they incorporate a range of study designs and address a broader range of research questions [26, 27].

We have conducted the scoping review in accordance with the five-stage approach outlined in the “Arksey and O`Malley [28] framework”: (1) Identifying the research question, (2) identifying the relevant studies, (3) study selection, (4) charting the data, and (5) collating, summarizing and reporting the results. Details about the search methods are described in a protocol that was registered in Open Science Frameworks [29].

### Identifying the research question

Based on the study aim, we constructed the following research question:

How does the research literature describe the content of rehabilitation models that support the transition phase from hospital to home for people discharged with ABI?

‘Content’ can be interpreted in multiple ways, and in this scoping review, we were particularly interested in thick descriptions of service models, such as organization forms, the agents involved, the context services were applied in, and any accompanying theoretical rationale for the models.

### Identifying the relevant studies

Initially, the first author (ME) conducted a limited search in the databases CINAHL and PubMed to identify index terms and text words relevant for the field of study. Based on this preliminary search, we developed a search strategy-chart (Table 1) (An overview of the full search strategy for all the included databases is provided in a supplementary appendix). In collaboration with a university librarian, the first author (ME) searched the following electronic databases: Medline, CINAHL, Amed, Cochrane, Embase, and Google Scholar. Hence, regular meetings between all four authors and the librarian generated an iterative search process where the choice of search terms was thoroughly adapted to each individual database.

**Table 1:** Search strategy-chart

#1 Population	#2 Context	#3 Concept
Acquired brain injury	“Home dwelling”	Hospital discharge
Cerebrovascular accident	“Living at home”	“Early supported discharge”
Traumatic brain injury	Home care	Team work
Brain Ischemia	“Community dwelling person”	Ambulatory care
Stroke survivor	Independent living	Transitional care
		Outpatient care
		Patient care
		Subacute care
		Rehabilitation
		Self-care (EO)
		Reablement mp.
		Community integration
		“Activities of daily living”

**Fig. 1:**
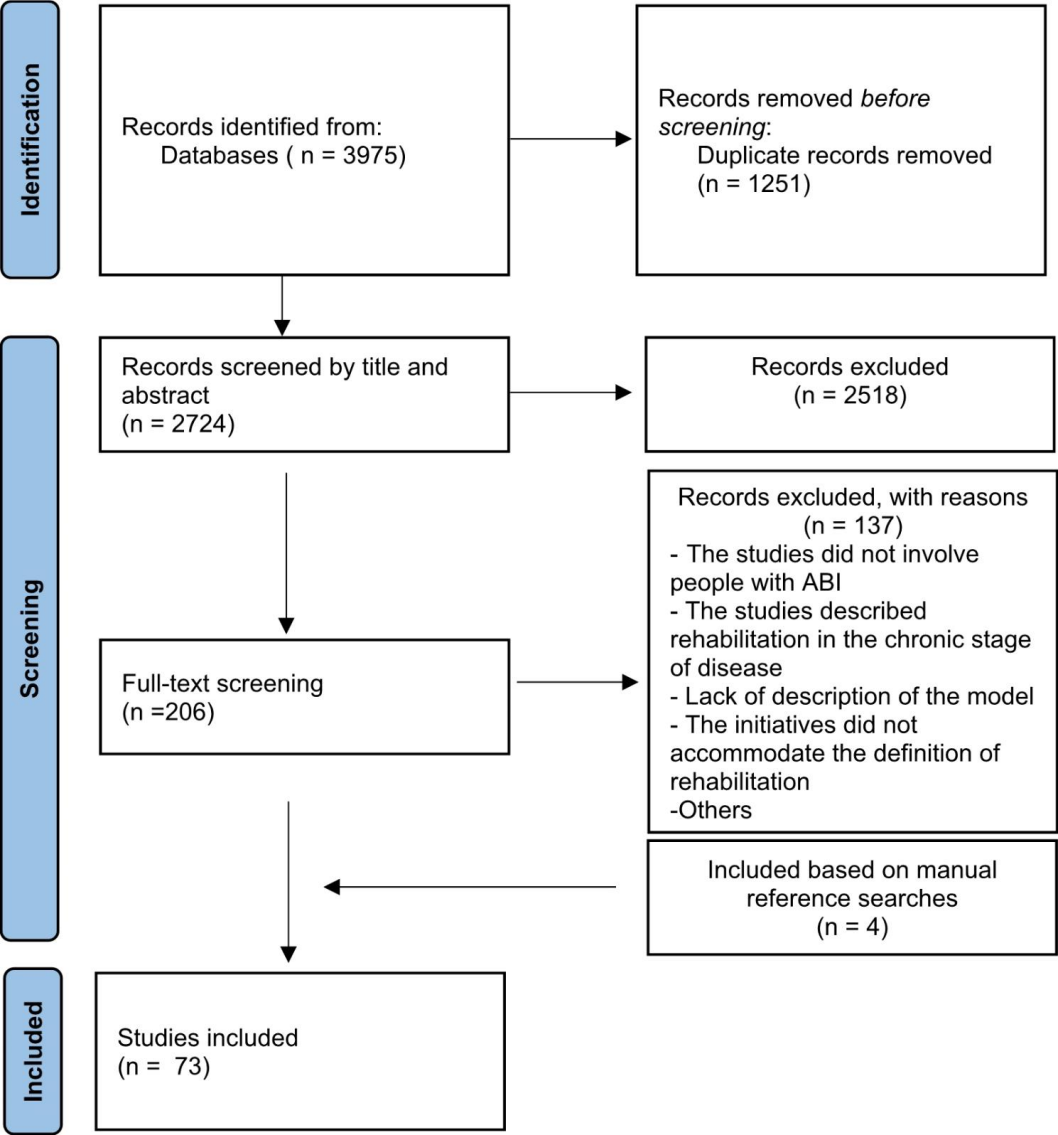
Flow diagram of the study selection process and outcomes. Modified PRISMA 2020 by Page MJ, McKenzie JE, Bossuyt PM, Boutron I, Hoffmann TC, Mulrow CD, et al. The PRISMA 2020 statement: an updated guideline for reporting systematic reviews. BMJ 2021;372:n71. 10.1136/bmj.n71

The search resulted in 3975 results (Fig. 1). To maintain transparency in the reporting of the process, we conducted consecutive memos. All peer-reviewed empirical studies, including qualitative and quantitative methodologies, were considered for inclusion. In the early 2000s, international consensus documents informed the development of an evidence-based model for early supported discharge services [30, 31]. Based on this, the timeframe for our search was limited to studies published after 2010. The selected timeframe was also set to provide research that could describe contemporary rehabilitation models. Studies published in English, Norwegian, Swedish and Danish were considered for inclusion. The searches were initiated in 2020, with a full update March 17th, 2023.

### Study selection

Two of the authors (ME, AG) performed the study selection independently and through iterative discussion meetings. Both authors, a physiotherapist and an occupational therapist, have research experiences within the field of rehabilitation and health care service research, respectively and the scoping review methodology. The two additional authors (CA and MN), both with competence within rehabilitation research, constituted an extended consultant team. The team met on a regular basis to discuss ambiguities, aiming for consensus. Table 2 provides a list of inclusion and exclusion criteria that guided the study selection process.

**Table 2:** Inclusion and exclusion criterion

	Inclusion criterion	Exclusion criterion
Type of studies	Peer reviewed empirical studies and reviews, masters and PhD thesis, protocols, conference-proceedings	Non-empirical studies
	All study designs, including qualitative and quantitative research and reviews	Political documents, opinion papers and books
	English, Norwegian, Swedish, Danish language	
User groups	User group should have an acute single-insult neurological injury (ABI)	Progressive neurological conditions
	Adult user group (> 18 years)	Patients with underlying brain injury, but where the focus is on other incidences or diagnoses
	Users that are being discharged home from hospital/rehabilitation ward in a subacute rehabilitation stage*	Chronic patients**
Type of interventions	Discharge rehabilitation service (in a subacute rehabilitation stage*)	Single-professional therapeutic interventions***
	Home-based or community-based rehabilitation	Long-term rehabilitation**
	Studies describing a service model	Interventions limited to specific part function-training***
		Psychometric analysis and studies validating assessment tools and test instruments
		In-hospital/institutional rehabilitation services*
		Care services that do not define as rehabilitation***

*We were particularly interested in models that target the transfer from in-hospital care to home. We have therefore limited the inclusion to involve studies that describe services that address this phase of rehabilitation pathway

**Services that address long-term follow up in a chronic phase are not included. There is no uniform consensus on the term chronic. In this study, a cut off for studies with an onset after 6 months after injury was set.

*** WHOs [115] definition of rehabilitation sounds ‘*a set of measures that assist individuals who experience, or are likely to experience, disability to achieve and maintain optimal functioning in interaction with their environments’*. This definition, in addition to the more refined Norwegian definition guides our interpretation of rehabilitation: ‘*Habilitation and rehabilitation must be based on the individual situation and goals of the individual patient and user. Habilitation and rehabilitation are targeted collaborative processes in various arenas between patient, user, relatives, and service providers. The processes are characterized by coordinated, coherent and knowledge-based actions. The purpose is that the individual patient and user, who have or are at risk of being restricted in their physical, mental, cognitive, or social functioning, should be given the opportunity to achieve the best possible functional- and coping ability, independence and participation in education and working life, socially and in the society’ (*https://helsedirektoratet.no/Retningslinjer/Hjerneslag*).* Compensating care services and traditional domiciliary care that do not aim for optimization of function, coping abilities and societal participation will therefore be excluded.

After the initial search, the reviewers searched for duplications (using both the software management tools Endnote and Rayyan, and eventually a manual screening of the references) or irrelevant publication sources (books, conference contributions, etc.) for exclusion, and 2724 articles were left for further screening.

In the first stage of inclusion and exclusion, two authors (ME and AG) screened all the titles and abstracts (n = 2724) to map out studies that were relevant to the research questions. The inclusion and exclusion criteria guided the screening process, which was first performed independently and blinded by the two reviewers. The sorting process was supported by Rayyan QCRI (Qatar Computing Research Institute: https://rayyan.qcri.org/welcome), a web app for exploring and filtering searches for eligible review studies. Conflicting decisions were discussed thoroughly until consensus was achieved. A total of 2518 studies were excluded in this process. The main reasons for exclusion in this process were as follows: (1) the studies did not involve people with ABI, (2) the studies described rehabilitation in the chronic stage of disease (defined as more than 6 months after injury), and (3) the studies did not include a description of a rehabilitation model.

In the second stage of study selection, the two reviewers performed a full text read of the temporary included articles (n = 206). The inclusion/exclusion-criterion list was adjusted and elaborated based on new insights acquired during the first screening stage, in accordance with the methodology [28]. The reviewers started with an individual and blinded review and further compared the decisions with each other. Conflicts were discussed to achieve consensus. A total of 137 studies were excluded at this stage, mainly because they did not include descriptions of the content of a rehabilitation model, or the initiatives did not accommodate the definition of rehabilitation as a multidisciplinary person-centered approach. For example, interventions that targeted delimited functional aspects, such as gait function, balance or hand locomotion, were not included in this scope. Neither were mono-professional services that omitted the interprofessional aspects of rehabilitation.

Additionally, four studies were included based on a manual reference list screening of the included studies, in accordance with Arksey and O’Malley [28]. Eventually, 73 studies were included for this scoping review. Figure 1 displays a Prisma flow diagram of the study selection strategy.

### Charting the data

According to Arksey and O’Malley [28], “charting the data” is a process similar to what would be called “data extraction” in a systematic review. Two of the authors (ME and AG) extracted data by using a predefined data charting form. We conducted an internal calibration exercise, pilot testing the chart, where the reviewers independently charted 12% of the included studies and then compared the results, striving for consensus. The additional studies were charted independently by the two reviewers. This is in line with Kastner et al. [27] suggestion to ensure the interreliability of the findings.

### Collating, summarizing, and reporting the results

Based on the charted data, we conducted a two-step analysis. First, we conducted a numerical analysis based on types of study designs, years of publication and the country in which the studies were conducted (Table 3). Second, we conducted a qualitative thematic analysis of the charted data, in line with Braun and Clarke’s six-phase model [32], aiming to synthesize descriptions of the different models. Initially, we read the included material multiple times to become familiar with the data. Second, we searched inductively for data that could provide insights into the content of the rehabilitation models. All the data that were perceived to be relevant, were coded with inductive (data-related) codes. Third, we arranged several joint discussions between the authors to identify patterns of similarities and differences, which enabled us to categorize the codes. Fourth, we reviewed and refined the codes and categories in an abductive manner, where interpretation by rehabilitation theory and research on health care services influenced the process. In the fifth step, we worked on identifying the specifics of each category as a process of generating main themes. Last, we drafted and wrote up the analysis, which resulted in the five types of models that are presented in Table 3 and the Results section. We used the software program NVivo 12 (QSR International) to organize the qualitative data analysis. The results are presented in a narrative summary.

**Table 3:** Summary of the included studies

Year of publication	2010–2011	2012–2013	2014–2015	2016–2017	2018–2019	2020–2021	2022- March 2023
	n = 6 [33–38]	n = 12 [21, 39–49]	n = 12 [31, 50–60]	n = 20 [61–80]	n = 4 [81–84]	n = 16 [85–100]	n = 3 [101–103]
**Patient group**	**Stroke**	**ABI**	**TBI**				
	n = 65 [21, 31, 33, 35, 36, 38–48, 50–64, 67–71, 73–92, 94–96, 98–103]	n = 7 [34, 37, 65, 66, 72, 93, 97]	n = 3 [84, 89, 92]				
**Region**	**Canada, USA**	**Scandinavia, Finland**	**Mid-Europe, GB**	**South Asia, China**	**Spain, Portugal**	**Australia**	**Iran**
	n = 16 [37, 38, 50, 54, 65, 67, 81, 84, 87–89, 92, 95, 97, 100, 102]	n = 13 [36, 48, 49, 51, 53, 57, 60, 66, 73, 74, 77, 80, 86]	n = 9 [31, 41, 45–47, 55, 62, 64, 103]	n = 16 [35, 39, 40, 43, 44, 56, 58, 59, 61, 63, 68, 82, 91, 96, 99, 101]	n = 2 [69, 78]	n = 4 [52, 83, 93, 94]	n = 1 [98]
**Model**	**Multidisciplinary home-based teams**	**Key coordinator**	**Trained family caregiver or lay health workers**	**Self-management programs**	**Pre-discharge planning**		
	n = 46 [21, 31, 33, 34, 36–38, 42, 45, 46, 48–51, 53, 54, 57, 60, 62–65, 67, 69–74, 76–80, 83, 86, 88, 89, 91, 93–95, 97, 100, 101, 103]	n = 8 [44, 47, 55, 58, 66, 81, 100–102]	n = 5 [56, 61, 75, 84, 92]	n = 11 [35, 39, 40, 43, 59, 68, 82, 87, 96, 99, 101]	n = 6 [41, 52, 72, 73, 88, 98]		

## Results

The numeric analysis shows that the research that describes models that support the transitional phase for people with ABI is distributed throughout the entire period from 2010 until March 2023, with a peak in 2016/2017. The majority of the studies concerned services for people with stroke (n = 66), while only three (n = 3) described services for people with traumatic brain injuries (TBIs). Seven studies (n = 7) involve a broader group of recipients classified as ABI, which can include both stroke patients, people with TBI and other insults. Service models from South Asia and China (n = 13), the Scandinavian countries (n = 13), USA and Canada (n = 16) were well represented in the included studies. Additionally, studies from Mid-Europe and Great Brittan also represent a solid proportion of the included studies (n = 9). We only identified one (n = 1) study from the Middle East (Iran), and no studies that described service models from African or South American countries that met our criteria for inclusion. Table 3 provides a systematic overview of the numeric analysis of this study.

Through our qualitative thematic analysis, we categorized the identified service organizations into five themes: (1) multidisciplinary home-based teams, (2) key coordinators, (3) trained family caregivers or lay health workers, (4) predischarge planning, and (5) self-management programs. Table 3 shows that the most frequently described service was the multidisciplinary home-based team model, which was described in 46 identified studies.

### Multidisciplinary home-based teams

The transition support model that was described most frequently was multidisciplinary home-based rehabilitation teams (n = 46). Descriptions are diverse, and there is a wide range of variety of such teams. Teams can be arranged by staff at the hospital [53, 79, 83, 86], by community health care services [94, 103], or by a collaboration where staff from both the hospital and community service collaborate within the same team [74, 78, 91]. A common feature of the model is that a multidisciplinary team carries out rehabilitation interventions in the care recipients’ homes. The most common professional groups to be involved were physiotherapists, occupational therapists, speech- and/or language therapists, social workers and registered nurses [42, 74, 79, 97, 101, 103]. Multidisciplinary collaboration was emphasized, and several articles reported that the service should involve a team coordinator [42, 46, 76, 95, 97]. Fisher et al. [46] stated that “a key care coordinator might be best to avoid multiple people thinking they are responsible for everything”. Regular team meetings and discussions were described as an essential part of these models [33, 38, 42, 53, 67, 76].

Although the duration and intensity of service delivery differed, the majority of studies agreed that the service interventions should start early, either in the hospital, or immediately after discharge home. Several described an intensive, short-term intervention from 4 to 6 weeks [31, 45, 53, 78, 86], others described services ranging from 8 to 12 weeks [54, 65, 91, 93], and Davoody et al. [80] and Markle-Reid et al. [38] described service interventions that lasted for 12 months. Both studies that described short length of stay [45] and long length of stay [80] reported that service users experienced a great need for follow-up and further support after ending interventions. In a Delphi study by Fisher et al. [46], a duration of 8 weeks was proposed for mild stroke survivors and 17 weeks for those with severe and complex deficits. However, consensus was not reached as standardization of intervention length conflicted with the principle of adjusting rehabilitation initiatives in accordance with individual needs.

Generally, descriptions of the service content, as well as contextually adjustments, were sparse in the included studies. Although a large proportion of the studies reported that the initiatives were based on the patients’ goals [31, 33, 48, 78, 81, 86, 93], descriptions of how this aspect were integrated in practice was not clear. Some studies stated that assessing what is important for the patient was central, but only Rafsten et al. [86] described that they used a particular assessment tool (the Canadian Occupational Performance Measure tool (COPM).

Another overarching goal for most of the described services was to facilitate community reintegration by empowering and supporting individuals in everyday activities, and to enhance self-care and independence. Approaches would involve ADL-training (activities of daily living-training) [38, 78, 86, 88], task specific approaches [74, 101], strategy-training techniques [88], problem-solving techniques and self-care strategies [79, 101]. However, we failed to identify distinct descriptions of how such interventions were carried out in practice.

Several studies described that their service model also involved family members and next of kin [63, 78, 80, 83, 86, 90, 100]. These studies emphasized that carers must be informed and educated about the situation and involved in the rehabilitation process, as well as addressing carers’ needs and supporting them in finding help in the community. A theoretical framework for conducting such family-based interventions was, however, not thoroughly described in any of the included studies.

Few of the included studies provided a theoretical or philosophical justification for their chosen interventions and approaches. The exceptions were Sritipsukho et al. [35] who described motor learning as theoretical foundation for the interventions and Lopez-Liria et al. [69] who based their approaches on a theoretical framework by Bobath. Others, such as Borg et al. [93], described the International Classification of Functioning, Disability and Health (ICF) as a foundation for the assessments by the team as their framework. Reunanen et al. [74] based their approaches on the theoretical framework of the “activating physiotherapy approach”.

The multidisciplinary home-based team model, such as the Early Supported Discharge-model (ESD) [31, 36, 45, 46, 51, 53, 57, 64, 86, 103] is particularly common in Scandinavian countries and Great Britain, but it is also described in Canada [37, 38, 50, 54, 67, 97], Australia [83, 93, 94] and the US [65, 81, 89]. Santana et al. [78] described how ESD was adapted and implemented in Portugal. The Portuguese model frequently used aids to carry out interventions, due to economic matters. Complementary descriptions of contextual features, such as population density and cultural and economic matters, were generally sparse.

### Key coordinator

Another service model discovered in this review was where a key coordinator or case manager was characterized as a fundamental part of the service provision. Examples of such models are the COMPASS-model from North Carolina, US [81, 100, 102], the stroke care coordinator (SCC-model) from the Maastricht area in the Netherlands [55], the longer-term stroke care model (LoTS) from the UK [47], the KORE and the ALT program from Denmark [66], and the transitional care model from Hong Kong [44, 58]. In a qualitative analysis of a key coordinator model, it was described that patients were truly satisfied with a designated case coordinator, as quoted: “I really find Karen (name of the coordinator) amazing. She really takes me by the hand” [66]. Most of the studies described nurses to undertake the role of a key coordinator [55, 58, 81, 100], although other health care professionals could also hold this position [47]. Other than that, we found no discussions about what competence or qualifications this coordinator should hold.

The studies described that a key-coordinator should be assigned as soon as possible after stroke onset, but the length of the different programs after discharge varied greatly. While Wong and Yeung [58] describe a duration of 4 weeks, Fens et al. [55] described a service model that lasted for 18 months. The interventions described in the key coordinator models are diverse, but often consist of outpatient visits, or telephone follow-up [58, 102]. The follow-up included patient information about risk factors, lifestyle, monitoring blood pressure [81], problem-solving techniques [47], and self-management strategies [58]. Additionally, identifying the caregivers’ needs [81, 100, 102], arranging health care facilities and physical aids, and recommending referrals to relevant therapy or other community resources were also central tasks [81, 102].

The coordinators’ direct involvement in rehabilitation tasks varied. In a Danish study, Glintborg and Hansen [66] described that the coordinator in the ALT program, which ran in smaller communities, had more face-to-face contact with the client and performing substantially more home visits after discharge. In the KORE program, which ran in the district capital, the coordinator mostly referred to client contact with others. Except for the Danish study, there was a lack of contextual descriptions that discussed geographic and demographic concerns. Fens et al. [55] described that the key-coordinator had a low threshold of consulting with the patient’s general practitioner (GP) or a multidisciplinary team in the community, and several other studies described collaborations with rehabilitation staff. However, there were few descriptions of how this collaboration was arranged.

### Trained family caregiver or lay health workers

Some of the identified studies described rehabilitation models that utilized persons other than health care professionals as the main executors of the rehabilitation initiatives, such as family caregivers and lay health workers. The ATTEND study from India [56, 61] and a study of e-health supported caregiver-mediated service in Australia [75] are examples of studies that describe trained family caregivers to conduct rehabilitation interventions. This way of arranging rehabilitation involved training and empowering the caregivers to deliver home-based rehabilitation. The rationale of such an arrangement was that it could potentially improve outcomes while using a minimum of health care resources, hence reducing health care costs [61].

The family-led services included in this review were initiated and administered from the hospitals, aiming to accelerate the patient’s hospital discharge. Health professionals trained and supervised the family caregivers by using a structured assessment and recommended rehabilitation package. The training programs were diverse but often described as short-term training during the hospital stay [61]. After discharge, the family-caregivers were the ones to follow up with the training at home [56, 75].

Interventions in the family-led services involved gait and gait-related mobility [75], patient information, repeated practice of task-specific activities, positioning, transfers, mobility, and discharge planning [56]. The included studies were based on a training manual [61] or a standardized exercise base [75]. However, individualization was described to be essential, and in the ATTEND study, the family caregiver, the patient, and the health care professionals arranged a joint goal-setting meeting [56]. However, there were no descriptions of the theoretical foundation for these approaches, nor was there a clear description of how the individualization could be operationalized.

The family-caregiver-led models in this study were supported by technological tools, such as a website where patients and family caregivers could choose exercise [56], a customized exercise app [75], or staff follow up thorough videoconferencing [75].

A rehabilitation model called Trabajadora de Salud was implemented in California inspired by a service model originally from Portugal and was administered by bilingual lay health workers in the community, referred to as trabajadoras [84, 92]. The intervention, which lasted for 90 days, involved home visits and previsits and reminder phone calls. Trabajadora de Salud followed a brief, empowering model focusing on brain injury education, referrals to community resources, help applying for resources and scheduling medical appointments, addressing basic needs, goal setting, assisting in health-care provider communication, and medical reconciliation providing empathy and validation of symptoms [92].

The organizational and technical aspects of the trained family caregiver services are thoroughly described; however, there is a lack of a theoretical framework to support the conduct of such a model, and descriptions of contextual features and theoretical foundations are lacking.

### Self-management programs

This review identified several studies (n = 11) that described self-management programs for rehabilitation, involving educational, and instructional approaches that aimed to support patients and caregivers in coping with life after injury with a minimum of staff involvement. Examples of such models are the Chinese 4 C, Home-Based Post-discharge program [101], the patient-Centered Self‐Management Empowerment Intervention (PCSMEI) caregiver [82], the social worker-led case management (SWCM) program from Michigan, USA [87], the Bal Ex Stroke model from Malaysia [59], and the rehabilitation shelter for homeless patients in Baltimore, USA [89].

Interventions were often initiated during the inpatient period [89, 96, 101] or immediately after the patients returned home after hospitalization [87] and could include individual home visits, group sessions [82, 99], follow-up phone-calls [87, 96, 101], and digitally supported aids, such as DVDs or apps [59, 68, 99]. Sritipsukho et al. [35] described an intervention strategy in Thailand that involved a home-based exercise program where the physiotherapist visited the patient only one hour per month for three months. Between the visits, patients were expected to carry out the training initiatives on their own.

The initiatives were designed to enhance personal resources, including promoting knowledge and self-care skills, enhancing self‐efficacy, and transferring self‐management knowledge and skills (training, goal setting, action taking, and resource utilization) [82, 101].

Contextual resources were used to establish a patient-centered network, which facilitated a collaborative relationship among the health care staff, patient, and family caregiver [82]. Several of the self-management models emphasized the importance of involving family members or informal carers in the approach [68, 82, 101]. The approach could also assess family members for stroke knowledge, worries and concerns about living with stroke patients [68, 82].

The studies mostly describe the organizational aspects and technical tools of these services. There is a lack of theoretical foundation for the involved measures.

### Predischarge planning

Some of the included studies described transition support models that mainly targeted predischarge planning, mostly initiated by hospital staff. We identified two variants of predischarge planning: in-hospital assessments and information [72, 98], and discharge-planning assessments and training at home [41, 52, 73, 88].

#### In-hospital assessments and information

A qualitative literature review [72] reported on experiences from several studies of discharge planning and summarized positive results for conducting family conferences in the hospital with patients who have suffered from a stroke. The involvement of the family or other caregivers was shown to be beneficial to the outcomes of the patient including coping, avoiding crises and support. The study also describes models that utilized an interactive website with resources and email contact with a nurse practitioner [72]. An Iranian study [98] described an in-hospital discharge planning where patients and care givers were provided with knowledge about the disease in addition to a booklet. Patients were followed-up through phone calls for two weeks post discharge.

#### Discharge-planning assessments and training at home

Drummond et al. [41] have described occupational therapy predischarge home visits in England, where they aimed to assess or practice activities of daily living in the home environment and to identify or address safety issues. Visits were generally conducted by an occupational therapist, with an occupational therapy or physiotherapy assistant. A trial protocol [88] also described a predischarge home visit where functional ability and barriers were assessed. Some predischarge models included training initiatives at home before the patient was discharged. A Danish study by Rasmussen et al. [73] described a model where representatives of a multidisciplinary hospital-based team trained the inpatient in their own home one to three times per week. Initiatives included physical exercises and activities of daily living. Hospital staff assessed the inpatient’s physical and cognitive abilities and provided them with temporary aids if needed. An Australian study [52] showed similarities in the STRENGTH model. In this model, the inpatient health care team (occupational therapist, physiotherapist, and speech pathologist) accompanied the clients at home, conducting therapy at home in the weeks leading up to discharge.

## Discussion

The results of this study provide knowledge about a wide range of diverse transition support models for people with ABI who are being discharged to their home. Despite the varied organization, we identified some key elements of the services, which may be applied as a common consensus for comprehensive transition support models for people with ABI.

### Organizational features

Most of the models that we identified through this scoping review, were organized in a hospital. However, some of the interdisciplinary team models were organized in primary care. Grimsmo et al. [104] argue that the feasibility of disease-specific clinical pathways in primary care is limited based on the low prevalence of patients with certain diagnoses in the context of primary care. This may call for services that are organized into specialist health care services, such as hospitals or corresponding enterprises, in the early phase of discharge. The feasibility of such models will, however, be challenged in rural areas with large geographical distances and less robust health care services. This may require rehabilitation efforts by the primary health care services in the communities.

There is evidence to support all five models identified in this scoping review. Early supported discharge (ESD) teams have been found to be successful in providing home-based rehabilitation to people with stroke [31, 105]. Such teams have been found to reduce the odds of death and dependency [21, 106], reduce the length of hospital stay [21, 107], result in faster improvement of overall disability [86], improved ADL ability [60], and a higher level of satisfaction with services compared to ordinary rehabilitation [21, 106, 108]. Qualitative studies have concluded that provision from an early supported discharge team was experienced as meaningful and adequate [77] and could reduce patients’ insecurity [7]. Although the benefits of ESD teams are well-documented in the field of stroke research, we only found one study that described ESD teams for the more general patient group of ABI [97]. Stroke is one of the most frequent diagnoses categorized as ABI [1] and represents the majority of research objects in our study (Table 3). Hence, there is a need to explore if services that are beneficial for stroke patients can be adapted to the more generalized group of patients with ABI. Models that are based on key-coordinator or case manager services are recommended by the ABIKUS Evidence Based Recommendations for Rehabilitation of Moderate to Severe Acquired Brain Injury [109]. Simpson et al. [110] argue that the complexity of the needs of people with TBI and their families requires both coordination among available services and support in managing the stress associated with unmet needs. Despite this, a systematic review [111] found limited evidence for case management services after brain injury. However, the lack of consensus of key coordinator roles and the diverse contexts of services, complicate any attempt to provide evidence. One must assume that the effects will vary based on the additional services there are to coordinate.

Services that mainly based their initiatives on training nonprofessionals, such as family caregivers or lay people, are in line with strategies emphasizing low-cost services and the economic sustainability of services. However, we have not found evidence to recommend such services compared to services delivered by professional rehabilitation staff.

Team models that are resource demanding were frequently represented in the Western countries, which tend to have well-developed health care services [112]. On the other hand, services that were delivered by nonprofessionals or self-management programs were mostly conducted in countries such as India [56, 61], China [68, 82], Malaysia [43, 59], and Thailand, but also in Australia [75], and the USA [87, 101]. This may indicate a socioeconomic inequity in service delivery from a global perspective, although it could also indicate that varied geographical dispersal or be related to ethnical and cultural factors and needs further investigation.

Although the studies included in this review recommend predischarge planning [72], it is unresolved whether such planning should be carried out as meetings at the hospitals or arranged as home visits that include assessment and training in the home environment. The evidence is diverse, and there is a paucity of applicable research on case management [111]. Investigations of predischarge occupational therapy found no difference in outcomes for people who do and do not receive predischarge home visits as part of their predischarge planning [113, 114]. Some of the predischarge services that involved a multidisciplinary team that carried out assessments and training in the patients’ home [52, 73], have similar features to the multidisciplinary rehabilitation teams described earlier. Therefore, we cannot draw a clear demarcation between the described service models, and one should consider the varied models as components of a continuous pathway.

Several of the included studies in this review involved combinations of the models that we identified. For example, the ABI TRS study from Australia [93], involves a combination of predischarge planning at the hospital and a multidisciplinary rehabilitation team in the home environment coordinated by a designated case manager. Some of the studies that describe ESD teams, also include a description of predischarge planning and coordination as part of the service. Chouliara et al. [31] emphasize the importance of considering such teams as “events in systems” and not as isolated from the broader organizational context. Both Simpson et al. [110] and Fisher et al. [46] call for services that combine case managers and multidisciplinary teams.

### Content of interventions and theoretical foundation

The described initiatives emphasized goal orientation and person/family centrality. Initiatives were described as targeting physical function, such as gait-related mobility, positioning, transferability, activities of daily life-function, and task-specific activities were commonly described in the included studies. Most of the included studies also emphasized the multidisciplinary aspect of rehabilitation; hence, they indicated that coordination and collaboration were central for the model. All these aspects are in line with how the WHO [115] defines central beliefs that are fundamental for rehabilitation:

*Functioning is central to health and well-being; it is integral to how a person is included and participates in meaningful activities and life roles.*

*Rehabilitation is person/family-centered; it is oriented around the specific needs and goals of the person and their family.*

*Rehabilitation is collaborative; it requires consultation with, and the active involvement of, the person and their family.*

Descriptions of which theoretical frameworks that supported the assessments and initiatives were sparse in the included studies. However, motor learning and activating physiotherapy were described as fundamental theoretical frameworks to support initiatives that targeted physical function. These theories are generally limited to interventions targeting an individual level and omit aspects that include a broader and comprehensive perspective that involve social integration and societal participation. The International Classification of Functioning, Disability and Health (ICF) model was described by Borg et al. [93] to serve as a fundament for assessments in their rehabilitation model. The ICF framework comprises the components of body functions and structures, activities, and participation [116], and is a worldwide accepted model providing a universal language for the description and classification of functioning. It is described by several as an appropriate framework in assessments, in addition to the development of rehabilitation strategies for people with complex needs [117–121].

In addition to physical functioning, problem-solving techniques, and self-management strategies, social activities, cognition, communication, and psycho-emotional status were also described as core features in the studies. Common issues for people suffering from ABI are psychological issues, such as depression, reduced attention and concentration, loss of memory or emotional issues [122, 123], and the literature recommends cognitive and behavior training as a central feature in rehabilitation for people with ABI [11]. However, the included studies in this scoping review do not describe any theoretical foundation for such initiatives. How such initiatives should be carried out in practice is clearly underreported in the research literature.

A family-centered perspective, where services aimed to support family members and next of kin, was identified in several of the included studies [59, 63, 68, 78, 80, 82, 83, 86, 90]. These studies focused on identifying and addressing carers’ needs and supporting them in their needs. Tramonti et al. [124] found the caregiver burden for family members of patients with ABI to exceed the cut-off score for possible burn-out.

In the included studies in this review, the family members’ role was described differently within the varied rehabilitation models. For example, in the self-management programs, family members were described to withhold their own emotional and practical needs, hence the rehabilitation models aimed to support them in coping with the new situation [59, 68, 82]. Contrary, in the family-led rehabilitation models, family members were primarily described to withhold a care-provider role, to support the patient with ABI [56, 61, 75], and descriptions on how to support their own needs were lacking.

Family members are potentially an important resource in the support of patients with ABI, and there is a need to explore how identification and support of family members’ needs can be an integrated part of a rehabilitation model.

### Contextual features: geographical patterns and cultural adaptations

The variety of different service models may indicate a lack of consensus on best practices for this population in the discharge phase of the rehabilitation trajectory. However, it may also be an expression of the need for contextual flexibility, in line with the metaphor of “one size does not fit all”. Despite the sparsely described contextual features in the studies, we did see some patterns that we will subsequently discuss.

The more complex and resource-intensive interprofessional teams were mostly described in literature published on studies from Scandinavia, the UK, Canada, and Australia. In one study from Portugal [78], researchers described a multidisciplinary team model, inspired by ESD-teams in Denmark, which they adjusted to the context of Portuguese health care services. Due to economic matters, they emphasized nonprofessional aides whom which they trained to provide rehabilitation initiatives instead of professional rehabilitation staff. This indicates that multidisciplinary teams are resource demanding, which may be the reason why they are more common in countries with robust welfare services.

Self-management services and services provided by trained family caregivers or lay health workers shared the common feature that they were depending on the labor force from other than health professionals, striving for economic sustainability. The identified studies that described such services were conducted in China, India, Malaysia, Thailand, Australia, and for the Latin American population in California, USA [35, 43, 56, 59, 61, 68, 75, 82, 84, 92]. Although we have no records of the cost of the services described in this study, it is noticeable that there are some geographical features that tend toward a pattern of low-cost services in Asian countries, and more resource demanding services in western countries. This may indicate a socioeconomic inequality in access to services, which has also been described earlier [125].

Some of the included studies indicate that there is a need to adjust services regarding population density, and that services delivered in rural areas require certain adjustments [30, 66, 67, 104]. Rural living has been demonstrated to affect citizens’ overall health status, and disparities in health between rural and urban individuals are an important global issue [67]. Individuals in rural settings often have poorer access to health care [126, 127], and poststroke rehabilitation services are a particular example [67]. However, further studies are needed to describe specific adjustments and adaptations to rural areas. Only one of the included studies highlighted cultural adjustments as central [92].

To utilize service research in a wide range of contexts, we encourage researchers to strive for more robust descriptions of geographical, demographical, and cultural contexts in future health care service research.

## Conclusions

This scoping review has evaluated a range of varied early discharge rehabilitation models for people with mild to moderate ABI worldwide. These models indicate that there is no global consensus about how to organize or how to conduct rehabilitation services in the subacute phase when patients are discharged to their home. However, it may also indicate that there is a need for contextual adjustments and adaptations of such services, depending on economy, geography, and culture. Our research also indicates that health care service research lacks robust and thorough descriptions that include such contextual features, which may limit the feasibility and transferability to diverse contexts. Scholars have debated that research often fails to describe interventions adequately, while mainly focusing on outcomes, rather than results of a descriptive nature [128]. Sparse descriptions may hamper implementation in practice and result in ‘research waste’. A systematic review explored 80 studies published in the journal *Evidence-Based Medicine* within one year, and found that only half of the studies could support clinicians to replicate the interventions [129]. This issue is, to our view, a consequence of a profound problem in health services research in general, which often is driven by researchers’ and journals’ interests of publishing outcomes, rather than providing research that is relevant for practice [130].

This scoping review showed that there is a need for further research in the field of early discharge services for people with ABI. The identified studies mostly target individual features and lack thorough descriptions of how to conduct rehabilitation that targets societal integration and participation. There is a need to develop a comprehensive theoretical framework to support such initiatives, and to explore how contextual features enable and limit service organization. These aspects are important to utilize the research results in appropriate contexts of practice. We would therefore like to put forward a proposal for more comprehensive descriptions of contextual and theoretical features for future rehabilitation research.

### Study strengths and limitations

This scoping review was based on 73 articles that describe diverse rehabilitation services for people with mild to moderate ABI. A large portion of the included studies were conducted with people following stroke and may not be appropriate for all people with ABI.

Our aim was to describe studies that targeted the subacute rehabilitation phase when patients are being discharged to their home. Therefore, we excluded studies that described rehabilitation models targeting the institutionalization phase and the chronic phase. We are, however, aware that this may have excluded studies that describe services that might also be relevant for the discharge home phase.

A wide range of studies that described mono-professional initiatives and delimited functional aspects were also excluded based on the exclusion criteria. This may have excluded studies that may include more detailed descriptions of service initiatives. A systematic review of such literature should be conducted to complement this study.

Moreover, studies were not examined for methodological rigor, which is considered to be irrelevant in the study design of scoping reviews [28]. The aim of this scoping review was to explore descriptions of service models, rather than study results (in line with the purpose of a scoping review [28]).

In this review we aimed to focus on rehabilitation models that supported the transition from hospital to home. However, as several of the primary studies lacked descriptions of whether patients were discharged from acute ward or in-patient rehabilitation, it was challenging for us to distinguish between these contexts, and we have therefore described explored the more general context of “hospital to home transition”. The results must be interpreted with an understanding that the patients may be discharged from various hospital settings.

The scoping review methodology opens for including gray literature [26, 28]. In this study, however, we limited the search to include only peer reviewed research literature. This decision was done to display a view of how the research field describes, or does not describe rehabilitation models in research. This must, however, be considered when interpreting our results, as there is a great possibility that there might exist a range of rehabilitation models that we have not been able to capture in this study. Further studies that also include gray literature, are needed to frame a complete picture of varied rehabilitation models for people with ABI in the transition from hospital to home.

According to Levac [26], scoping reviews may benefit from consulting with relevant stakeholders. In the process of conducting this scoping review, the researchers arranged a digital webinar where approximately 30 stakeholders within the field of rehabilitation for persons with ABI (patients, clinicians, program managers, and researchers) were invited to participate. The search strategy and preliminary results were presented, and the stakeholders were invited to comment on the process. However, no new insights were identified based on the event. The digital form of such event, including a relatively large group of people who did not know each other, may be part of the explanation of why this event did not offer any new insights.

To strengthen the validity of the study, we engaged a librarian to support the search process. A study strength was that two of the authors reviewed the articles, and continuously engaged in discussions with each other and the extended group of authors to achieve consensus. However, this process is fundamentally interpretive, and we are aware that our study selection and inclusion may not be exhaustive.

The study is presented in line with the PRISMA Extension for Scoping Reviews (PRISMA-ScR), guideline for reporting scoping reviews [131].

## Electronic supplementary material

Below is the link to the electronic supplementary material.

Supplementary Material 1

## Data Availability

Data is retrieved from published articles. All included articles are listed in the manuscript (Table 3) and supported with full references in the reference list.

## List of abbreviations

ABI: Acquired brain injury
ABIKUS: Acquired Brain Injury Knowledge Uptake Strategy
ADL: Activities of daily living
COMPASS: Cardiovascular Outcomes for People Using Anticoagulation Strategies
COPM: Canadian Occupational Performance Measure tool
ESD: Early Supported Discharge-model
GP: General practitioner
ICF: International Classification of Functioning, Disability and Health
LoTS: Longer-term stroke care model
PCSMEI: Patient-Centered Self‐Management Empowerment Intervention
PRISMA-ScR: Preferred Reporting Items for Systematic reviews and Meta-Analyses extension for Scoping Reviews
QCRI: Qatar Computing Research Institute
QSR: Qualitative research software
SCC: Stroke care coordinator
SWCM: Social worker-led case management
TBI: Traumatic brain injuries
TRS: Transitional Rehabilitation Service
WHO: World Health Organization
