# Supplementary material for: Rehabilitation models that support transitions from hospital to home for people with acquired brain injury (ABI): a scoping review
Source: BMC Health Serv Res. 2023 Jul 31;23:814. doi: 10.1186/s12913-023-09793-x (PMC10388520; doi:10.1186/s12913-023-09793-x)
Supplement: Supplementary file 1 — Supplementary Material 1 [file 12913_2023_9793_MOESM1_ESM.docx]

***Medline (Ovid)***

| #1 | exp brain injury/ or exp cardiovascular accident/ or exp traumatic brain injury/or exp brain ischemia/ or exp stroke survivor/ or exp stroke |
| --- | --- |
| #2 | Home ADJ5 dwelling/ or living at home/ or home ADJ5 care/ or community ADJ5 dwelling/ or exp independent living |
| #3 | Hospital adj5 discharge/ or early supported discharge/ or teamwork/ or ambulatory care/ or Transitional care/ or outpatient care/ or patient care/ or subacute care/ or exp. Rehabilitation /or community ADJ5 integration/ or “activities of daily living”/ or Selfcare/ or Reablement/ or re-ablement |
| #4 | #1 AND #2 AND 3 |
| #5 | (2010:2023) |
| #6 | #4 AND #5 |

***Embase (Ovid)***

| #1 | Exp acquired brain injury/ or exp cerebrovascular accident/ or exp traumatic brain injury/ or exp brain ischemia/ or exp stroke survivor |
| --- | --- |
| #2 | “home dwelling” / or exp living at home/ or exp home care/ or exp community dwelling person/ or exp independent living |
| #3 | Exp hospital discharge/ or exp early supported discharge/ or exp teamwork/ or ambulatory care/ or transitional care/ or outpatient care/ or exp patient care / or exp subacute care/ or (exp rehabilitation or narrower)/ or community integration/ or “activities of daily living”/ or selfcare /or reablement/ or re-ablement |
| #4 | #1 AND #2 AND #3 |
| #5 | (2010-2023) |
| #6 | #4 AND #5 |

***Amed (Ovid)***

| #1 | exp brain injury/ or exp cardiovascular accident/ or exp traumatic brain injury/or exp brain ischemia/ or exp stroke survivor/ or exp stroke |
| --- | --- |
| #2 | Home ADJ5 dwelling/ or living at home/ or home ADJ5 care/ or community ADJ5 dwelling/ or exp independent living |
| #3 | Hospital adj5 discharge/ or early supported discharge/ or teamwork/ or ambulatory care/ or Transitional care/ or outpatient care/ or patient care/ or subacute care/ or exp. Rehabilitation /or community ADJ5 integration/ or “activities of daily living”/ or Selfcare/ or Reablement/ or re-ablement |
| #4 | #1 AND #2 AND 3 |
| #5 | (2010:2023) |
| #6 | #4 AND #5 |

***Cinahl (EBSCO)***

| #1 | exp MH Brain Injurie /or exp MH stroke /or MH stroke patients /or brain ischemia /or neurological impairment* |
| --- | --- |
| #2 | “home N5 dwelling/ or Living W5 home /or exp home health care /or community dwelling /or independent living /or MH community living |
| #3 | Early supported discharge /or MH early patient discharge /or rehabilitation W5 team* /or exp MH patient discharge /or “interprofessional team” /or “post discharge /or MH ambulatory care /or exp MH continuity of patient care /or transition N5 home/or care trajectory* /or MH transitional care /or outpatient N5 rehabilitation /or extended stroke unit* /or ext MH home rehabilitation /or MH (Transfer, Discharge) /or ext MH rehabilitation /or community N5 integration /or MH “activities of daily living” /or “self management” /or reablement /or re-ablement /or stroke management /or stroke care /or restorative care |
| #4 | #1 AND #2 AND #3 |
| #5 | 2020:2023 |
| #6 | #4 AND #5 |

***Cochrane Library***

| #1 | MeSH descriptor: [brain injury] expl /or MeSH descriptor: [stroke] expl /or “stroke survivor” /or MeSH descriptor: [cerebrovascular disorders] expl |
| --- | --- |
| #2 | MeSH descriptor: [independent living] expl /or community dwelling or/ MeSH descriptor: [self-management] |
| #3 | “Early supported discharge /or “rehabilitation team*” /or MeSH descriptor: [patient discharge] expl /or MeSH descriptor: [subacute care] expl /or MeSH descriptor: [patient care team] expl /or MeSH descriptor: [home care services] expl /or MeSH descriptor: [ambulatory care] expl /or MeSH descriptor: [continuity of patient care] expl /or MeSH descriptor: [transition care] /or MeSH descriptor: [rehabilitation] expl /or “community integration /or MeSH descriptor: [activities of daily living] expl /or “restorative care /or “stroke care” /or re?ablement /or “stroke management |
| #4 | #1 AND #2 AND #3 |
| #5 | (2020:2023) |
| #6 | #4 AND #5 |

***Google Scholar***

brain injury | stroke | traumatic brain injury | headinjury | ABI | TBI |transitional care | subacute care| home care | rehabilitation | model | framework | service | program | home dwelling | home living | home rehabilitation | community integration
